# Joint Effects of Long-Term Exposure to Ambient Fine Particulate Matter and Ozone on Asthmatic Symptoms: Prospective Cohort Study

**DOI:** 10.2196/47403

**Published:** 2023-08-03

**Authors:** Jiahong Xu, Yan Shi, Gongbo Chen, Yanfei Guo, Weiling Tang, Cuiling Wu, Shuru Liang, Zhongguo Huang, Guanhao He, Xiaomei Dong, Ganxiang Cao, Pan Yang, Ziqiang Lin, Sui Zhu, Fan Wu, Tao Liu, Wenjun Ma

**Affiliations:** 1 Department of Public Health and Preventive Medicine School of Medicine Jinan University Guangzhou China; 2 Shanghai Municipal Centre for Disease Control and Prevention Shanghai China; 3 School of Public Health and Preventive Medicine Monash University Victoria Australia; 4 School of Public Health Guangdong Pharmaceutical University Guangzhou China; 5 Shanghai Medical College Fudan University Shanghai China

**Keywords:** asthma, joint association, ozone, PM2.5, population attributable fraction, air pollution, long-term exposure

## Abstract

**Background:**

The associations of long-term exposure to air pollutants in the presence of asthmatic symptoms remain inconclusive and the joint effects of air pollutants as a mixture are unclear.

**Objective:**

We aimed to investigate the individual and joint associations of long-term exposure to ambient fine particulate matter (PM_2.5_) and daily 8-hour maximum ozone concentrations (MDA8 O_3_) in the presence of asthmatic symptoms in Chinese adults.

**Methods:**

Data were derived from the World Health Organization Study on Global Ageing and Adult Health (WHO SAGE) cohort study among adults aged 50 years or older, which was implemented in 1 municipality and 7 provinces across China during 2007-2018. Annual average MDA8 O_3_ and PM_2.5_ at individual residential addresses were estimated by an iterative random forest model and a satellite-based spatiotemporal model, respectively. Participants who were diagnosed with asthma by a doctor or taking asthma-related therapies or experiencing related conditions within the past 12 months were recorded as having asthmatic symptoms. The individual associations of PM_2.5_ and MDA8 O_3_ with asthmatic symptoms were estimated by a Cox proportional hazards regression model, and the joint association was estimated by a quantile g-computation model. A series of subgroup analyses was applied to examine the potential modifications of some characteristics. We also calculated the population-attributable fraction (PAF) of asthmatic symptoms attributed to PM_2.5_ and MDA8 O_3_.

**Results:**

A total of 8490 adults older than 50 years were included, and the average follow-up duration was 6.9 years. During the follow-up periods, 586 (6.9%) participants reported asthmatic symptoms. Individual effect analyses showed that the risk of asthmatic symptoms was positively associated with MDA8 O_3_ (hazard ratio [HR] 1.12, 95% CI 1.01-1.24, for per quantile) and PM_2.5_ (HR 1.18, 95% CI 1.05-1.31, for per quantile). Joint effect analyses showed that per equal quantile increment of MDA8 O_3_ and PM_2.5_ was associated with an 18% (HR 1.18, 95% CI 1.05-1.33) increase in the risk of asthmatic symptoms, and PM_2.5_ contributed more (68%) in the joint effects. The individual PAFs of asthmatic symptoms attributable to PM_2.5_ and MDA8 O_3_ were 2.86% (95% CI 0.17%-5.50%) and 4.83% (95% CI 1.42%-7.25%), respectively, while the joint PAF of asthmatic symptoms attributable to exposure mixture was 4.32% (95% CI 1.10%-7.46%). The joint associations were greater in participants with obesity, in urban areas, with lower family income, and who used unclean household cooking fuel.

**Conclusions:**

Long-term exposure to PM_2.5_ and MDA8 O_3_ may individually and jointly increase the risk of asthmatic symptoms, and the joint effects were smaller than the sum of individual effects. These findings informed the importance of joint associations of long-term exposure to air pollutants with asthma.

## Introduction

Asthma is a heterogeneous disease defined by a history of respiratory symptoms (eg, wheezing, shortness of breath, chest tightness, and cough) that vary over time and in intensity, accompanied by variable expiratory airflow limitation [1]. Asthma could affect people of all ages worldwide. The Global Burden of Disease (GBD) study in 2019 estimated that there were 262 million people worldwide affected by asthma, with a 15.7% increase in prevalence since 2010 [2].

The complexity of asthma is particularly challenging, and increasing evidence has linked air pollution exposures to the incidence of asthma. There is ample evidence that supports the association of short-term exposure to air pollution and morbidity of asthma [3,4]. However, the associations between long-term exposure to air pollution and asthma remain inconclusive [5]. Some studies reported positive associations of long-term exposure to air pollutants with the incidence of asthma [6-11], while others reported null or negative associations [12-15]. This inconsistency may be related to different populations, exposure assessment approaches for air pollution, and study designs among previous studies. For example, several studies were conducted in children, in which the role of chronic air pollution exposure in the development of childhood asthma has been well demonstrated [4,16], while in some studies conducted in adults, the role of air pollution in adult-onset asthma was inconclusive [6,7,10,11]. It was suggested that childhood-onset and adult-onset asthma are 2 distinct asthma phenotypes that have different clinical, biological, and genetic characteristics [17]. The associations of air pollution with adult-onset asthma should not be extrapolated from studies in children and should be particularly investigated. In addition, more studies that investigated the chronic effects of air pollution on asthma were cross-sectional studies, and few were prospective studies [18]. Those results from cross-sectional studies may be biased due to recall bias and confounding bias. Therefore, we believe more prospective studies on adults are needed.

Although studies on the long-term effects of air pollution on asthma in adults are few and inconsistent, several previous studies showed a positive association between air pollution and other chronic respiratory diseases such as chronic obstructive pulmonary disease (COPD). For example, a 15-year population cohort study in Canada found positive associations of COPD with ambient fine particulate matter (PM_2.5_) and O_3_ [19]. A systemic review and meta-analysis showed that per 10 μg/m^3^ increase in PM_2.5_ is associated with an increased incidence of COPD (hazard ratio [HR] 1.18, 95% CI 1.13-1.23) [20]. These studies suggested the plausibility of the association between air pollution and asthma.

It is increasingly recognized that people in their daily lives do not get exposed to a single air pollutant but rather to multiple air pollutants as a mixture that altogether may impact health. The idea of coexposures to multiple pollutants has been articulated as part of the exposome concept [21], and the scientific community and the US Environmental Protection Agency are moving toward a multipollutant approach to quantify the health consequences of air pollution mixtures as a whole [22]. A prospective cohort study from Australia reported a significant joint association of 5 air pollutants (PM_10_, PM_2.5_, CO, NO*_x_*, and SO_2_) with COPD, although they did not find significant associations of COPD with several pollutants in the multiple pollutant model [12], suggesting the importance of assessing joint effects of air pollutants as a whole. However, to date, very few studies have estimated the joint effects of air pollutants on the risk of asthma.

China is one of the most popular countries with a large number of patients with asthma and has serious air pollution. The GBD 2019 study estimated that there were about 24.8 million patients with asthma across China in 2019 [2]. The annual average PM_2.5_ concentration across China was as high as 77.05 μg/m^3^ in 2013 [23]. To tackle severe air pollution, Chinese governments have implemented a series of rigorous air pollution control policies in the past decade [24]. Although the national average PM_2.5_ concentration across China in 2021 has been significantly reduced to 30 μg/m^3^, it is still 6 times higher than the recommended air quality guidelines (5 μg/m^3^) by World Health Organization (WHO) (2021). In addition, these control policies in air pollution inappropriately reduce NO*_x_* rather than the volatile organic compounds, which may lead to an increase in the ambient ozone (O_3_) [25]. The national population-weighted mean concentrations of maximum daily 8-hour average ozone (MDA8 O_3_) increased from 89.34 μg/m^3^ in 2013 to 100.96 μg/m^3^ in 2019 [26]. Currently, both ambient PM_2.5_ and O_3_ have become the major air pollutants across China. However, the individual and joint associations of long-term exposure to PM_2.5_ and O_3_ with asthma in adults remain unknown.

To fill in these research gaps, this study implemented a national cohort study in China to estimate both individual and joint associations of long-term exposure to PM_2.5_ and O_3_ with risk of asthmatic symptoms and calculated the population-attributable fraction (PAF) of asthmatic symptoms attributable to PM_2.5_ and O_3_ in Chinese adults aged older than 50 years.

## Methods

### Study Design and Population

The World Health Organization Study on Global Ageing and Adult Health (WHO SAGE) is a cohort study that was conducted in 1 municipality (Shanghai) and 7 provinces (Guangdong, Hubei, Jilin, Shaanxi, Shandong, Yunnan, and Zhejiang) in China by using a stratified multistage random cluster sampling among adults aged older than 18 years and older from 2007 to 2018 [27,28]. A total of 18,673 participants were recruited at baseline in the first wave (2007-2010) conducted through a face-to-face household interview and were followed up during 2014-2015 and 2017-2018, respectively. In each round of follow-up investigation, new participants were added to enlarge the sample size. Eventually, a total of 18,673 participants were included in the research. Participants were excluded from this study if they were lost to follow-up (n=8053), reported asthmatic symptoms at baseline (n=420), unavailability of data for follow-up (n=33), without information about asthma (n=676), were younger than 50 years (n=728), and missing information on key covariates (n=273). Finally, a total of 8490 participants were included in the analysis (Figure S1 in Multimedia Appendix 1).

### Ethics Approval

This study was approved by the Ethics Committee of the Chinese Centre for Disease Control and Prevention. Informed consent was obtained from each participant before the interview. Individually identifiable information was anonymous. WHO SAGE was approved by the World Health Organization’s Ethical Review Board (RPC146), in China, SAGE wave1 was approved by the Chinese Center for Disease Control and Prevention (200601), and SAGE wave 2-3 was approved by Shanghai Municipal Center for Disease Control and Prevention (2014-8, 2018-1).

### Assessment of Environmental Exposures

Daily 8-hour maximum ozone concentrations (MDA8 O_3_) with a spatial resolution of 7×7 km across China during 2008-2018 were estimated by an iterative random forest model, which were derived from ground monitoring ozone data, satellite-derived ozone column amount, various meteorological information, normalized difference vegetation index, fractional urban cover, and elevation data. The random forest model showed sample-based and site-based cross-validation *R*^2^ of 0.84 and 0.79, respectively, indicating the high accuracy of estimation of daily MDA8 O_3_ [29]. The annual average MDA8 O_3_ was calculated using the daily data in each grid. Annual mean PM_2.5_ concentration was measured by a satellite-based spatiotemporal model with a spatial resolution of 1×1 km across China, which was established by the Atmospheric Composition Analysis Group. The *R*^2^ value for the spatiotemporal model was 0.81 [30]. The annual average temperature was obtained from ERA5 [31].

We first calculated the annual mean exposure to MDA8 O_3_, PM_2.5_, and temperature in each calendar year (from the first day to the last day of a year) at a spatial grid across China. Then, the environment exposure data were assigned to each participant based on latitude and longitude of residence address, which ensured the accuracy of exposure information if the participant changed address. One-year exposure was regarded as an indicator of long-term exposure level and treated as a time-varying variable [32,33].

### Outcome Measurement

Participants who were diagnosed with asthma by a doctor prior to the interview or taking asthma-related therapies within the past 12 months prior to the interview were recorded as having asthmatic symptoms. Participants were also defined as cases with asthmatic symptoms based on an algorithm about self-reported symptom-based questions within the past 12 months prior to the survey that is listed in Table S1 in Multimedia Appendix 1 [34]. The outcome was collected at baseline and at each round of follow-up, and the new asthmatic symptom was defined as the first ever met the condition listed above during follow-up.

### Covariates

Covariates in this study included age (50-64 years and ≥65 years), sex (male and female), urbanicity (urban and rural), region (southern and northern), smoking status (ever and never), drinking status (ever, never), BMI (kg/m^2^) (normal [≤23.9], overweight [24.0-27.9], and obese [≥28]), marriage status (married and unmarried), family income (low and high), major type of household cooking fuel (clean fuel and unclean fuel), educational level (no formal education, primary school, and middle school or higher), physical activity (low, middle, and high), fruit intake (sufficient and insufficient), vegetable intake (sufficient and insufficient), air pollution–related occupations (related and not related), and ambient temperature (Table S1 in Multimedia Appendix 1).

### Statistical Analysis

#### Individual Associations of Air Pollutants With the Presence of Asthmatic Symptoms

A Cox proportional hazards model using time-varying covariates and coefficients [35] was used to estimate the HRs and 95% CI on the presence of asthmatic symptoms for each quartile increment in the pollutant concentration in the single-pollutant model. We also explored the nonlinear effects by applying a natural spline with 3 degrees of freedom in the model. We applied a series of subgroups analysis to examine the potential modifications of some characteristics.

Based on the above estimates, we used the following formula (equation 1) to calculate the individual PAF of asthmatic symptoms attributable to PM_2.5_ and MDA8 O_3_ [36]:



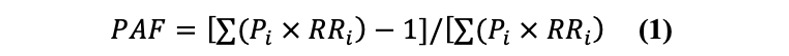



where *P_i_* indicates the proportion of the population in exposure category “*i*” (using each PM_2.5_ and MDA8 O_3_ concentration quantile as the category) and *RR_i_* represents HR of exposure category “*i*.”

#### Joint Associations of Air Pollutants With the Presence of Asthmatic Symptoms

We first tested the collinearity among air pollutants and covariates using the Variance Inflation Score, and the results illustrated that the low collinearity with the Variance Inflation Scores of the multipollutant model among variables was less than 10 (Table S2 in Multimedia Appendix 1).

A quantile g-computation model was used by using a Cox proportional hazards model as an underlying model to estimate the joint effects of PM_2.5_ and MDA8 O_3_. We estimated the joint effect of air pollutants for an equal quartile increment of each pollutant in the model. The quantile g-computation model first transformed each pollutant into a categorical variable, coded as 0, 1, 2, 3, and then estimated associations with health outcomes when all pollutants change 1 unit. The model also calculated the weight of each pollutant, if all the exposure pollutants have the same direction, interpreting as the proportion of the effect and sum to 1. If the exposure pollutants have different directions of effect, the weights are interpreted as a proportion of the positive or negative effect, and sum to 2 [37]. Moreover, the quantile g-computation model was also conducted in subgroups. The between-group variability was tested using a 2-sample *z* test [38], and *P* values calculated by 2-by-2 comparisons between multiple groups were corrected using the Bonferroni method. The formula (equation 2) for the between-group variability test is as follows:



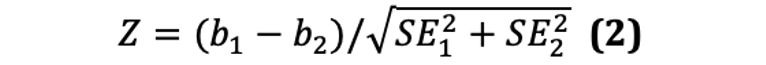



where *b*_1_ and *b*_2_ are the estimated effects of 2 groups, and *SE*_1_^2^ and *SE*_2_^2^ are the standard errors of the estimated effects of 2 groups [38].

Based on the above estimates, we calculated the individual and joint PAFs of asthmatic symptoms attributable to PM_2.5_ and MDA8 O_3_ [36]. We used the following formula (equation 3) to calculate the joint PAF:



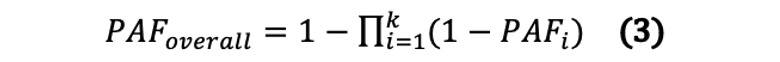



where *PAF_overall_* represents the effect values of all risk factors, *PAF_i_* is the PAF for exposure category “*i*.” A variance-covariance matrix of the parameters from the regression model was used to estimate the 95% CI of joint PAF by randomly generating 10,000 PAFs from the normal distribution.

### Sensitivity Analysis

Sensitivity analysis adjustment for different confounders was used to test the robustness of our findings. For some confounders with serious missing such as physical activity, fruit intake, and vegetable intake, sensitivity analyses were conducted only in participants with completed data.

All analyses were conducted by R software (version 4.1.3; R Development Core Team). All tests were 2-tailed, and *P*<.05 was considered statistically significant.

## Results

### Characteristics of Study Participants

A total of 8490 participants were finally included in this analysis, with a mean of 6.9 (SD 2.3) years of follow-up and an overall 58,737 person-years of follow-up. Among the total participants, 2950 (34.75%) were older than 64 years, 4066 were (47.89%) males, 4867 (57.43%) resided in rural areas, 5259 (61.94%) lived in Southern China, and 4641(54.66%) used unclean energy as the major household cooking fuel. At the follow-up, 586 (6.9%) participants were diagnosed with asthmatic symptoms (Table 1).

The mean PM_2.5_ concentration was 42.85 (SD 12.74) μg/m^3^, and the 25%, 50%, and 75% quantiles were 31.70 μg/m^3^, 41.30 μg/m^3^, and 53.10 μg/m^3^, respectively. The mean MDA8 O_3_ concentration was 94.19 (SD 5.62) μg/m^3^, and the 25%, 50%, and 75% quantiles were 89.12 μg/m^3^, 93.73 μg/m^3^, and 97.29 μg/m^3^, respectively. PM_2.5_ was positively correlated with ambient MDA8 O_3_ (*r*=0.663, *P*<.001) and ambient temperature (*r*=0.090, *P*<.001). Ambient MDA8 O_3_ was also positively correlated with ambient temperature (*r*=0.428, *P*<.001; Table S3 in Multimedia Appendix 1).

**Table 1:** Characteristics of study participants in the World Health Organization Study on Global Ageing and Adult Health cohort study in China.

Categories		Total participants (N=8490)	Participants with asthmatic symptoms during the follow-up	
			No (n=7904)	Yes (n=586)
MDA8 O_3_ (μg/m^3^), mean (SD)		94.19 (5.62)	94.18 (5.64)	94.12 (5.46)
PM_2.5_ (μg/m^3^), mean (SD)		46.20 (12.74)	46.02 (12.64)	46.64 (14.25)
**Age (years), n (%)**		61.89 (8.57)	61.71 (8.52)	64.31 (8.9)
	50-64	5540 (65.25)	5225 (66.11)	315 (53.8)
	≥65	2950 (34.75)	2679 (33.89)	271 (46.2)
**Gender, n (%)**				
	Male	4066 (47.89)	3780 (47.82)	286 (48.8)
	Female	4424 (52.11)	4124 (52.18)	300 (51.2)
**Urbanicity, n (%)**				
	Rural	4876 (57.43)	4606 (58.27)	270 (46.1)
	Urban	3614 (42.57)	3298 (41.73)	316 (53.9)
**Region, n (%)**				
	Southern China	5259 (61.94)	4943 (62.54)	316 (53.9)
	Northern China	3231 (38.06)	2961 (37.46)	270 (46.1)
**Marital status, n (%)**				
	Married	7340 (86.45)	6861 (86.80)	479 (81.7)
	Unmarried	1150 (13.55)	1043 (13.20)	107 (18.3)
**BMI (kg/m^2^), n (%)**				
	Normal weight (≤23.9)	4683 (55.16)	4359 (55.2)	324 (55.3)
	Overweight (24-27.9)	2772 (32.65)	2587 (32.73)	185 (31.6)
	Obese (≥28)	1035 (12.19)	958 (12.12)	77 (13.1)
**Major type of household cooking fuel, n (%)**				
	Clean	4641 (54.66)	4308 (54.50)	333 (56.8)
	Unclean	3849 (45.34)	3596 (45.50)	253 (43.2)
**Smoking status, n (%)**				
	Ever	2819 (33.20)	2609 (33.01)	210 (35.8)
	Never	5671 (66.80)	5295 (66.99)	376 (64.2)
**Drinking status, n (%)**				
	Ever	2716 (31.99)	2526 (31.96)	190 (32.4)
	Never	5774 (68.01)	5378 (68.04)	396 (67.6)
**Family income, n (%)**				
	High	4306 (50.72)	4008 (50.71)	298 (50.8)
	Low	4184 (49.28)	3896 (49.29)	288 (49.2)
**Educational level, n (%)**				
	No formal education	3393 (39.97)	3154 (39.90)	239 (40.8)
	Primary school	1802 (21.22)	1662 (21.03)	140 (23.9)
	Middle school or higher	3295 (38.81)	3088 (39.07)	207 (35.3)
**Fruit intake, n (%)**				
	Sufficient	1259 (14.83)	1151 (14.56)	108 (18.4)
	Insufficient	6699 (78.90)	6253 (79.11)	446 (76.1)
	Missing	532 (6.27)	500 (6.33)	32 (5.5)
**Vegetable intake, n (%)**				
	Sufficient	5828 (68.64)	5407 (68.41)	421 (71.8)
	Insufficient	2417 (28.47)	2266 (28.67)	151 (25.8)
	Missing	245 (2.89)	231 (2.92)	14 (2.4)
**Air pollution–related occupations, n (%)**				
	Related	1045 (12.31)	950 (12.02)	95 (16.2)
	Not related	6358 (74.89)	5945 (75.21)	413 (70.5)
	Missing	1087 (12.80)	1009 (12.77)	78 (13.3)
**Physical activity, n (%)**				
	High	4025 (47.41)	3749 (47.43)	276 (47.1)
	Middle	1737 (20.46)	1625 (20.56)	112 (19.1)
	Low	1611 (18.98)	1495 (18.91)	116 (19.8)
	Missing	1117 (13.15)	1035 (13.10)	82 (14)

### Individual Effects of Long-Term Exposure to MDA8 O_3_ and PM_2.5_ in the Presence of Asthmatic Symptoms

**Figure 1:**
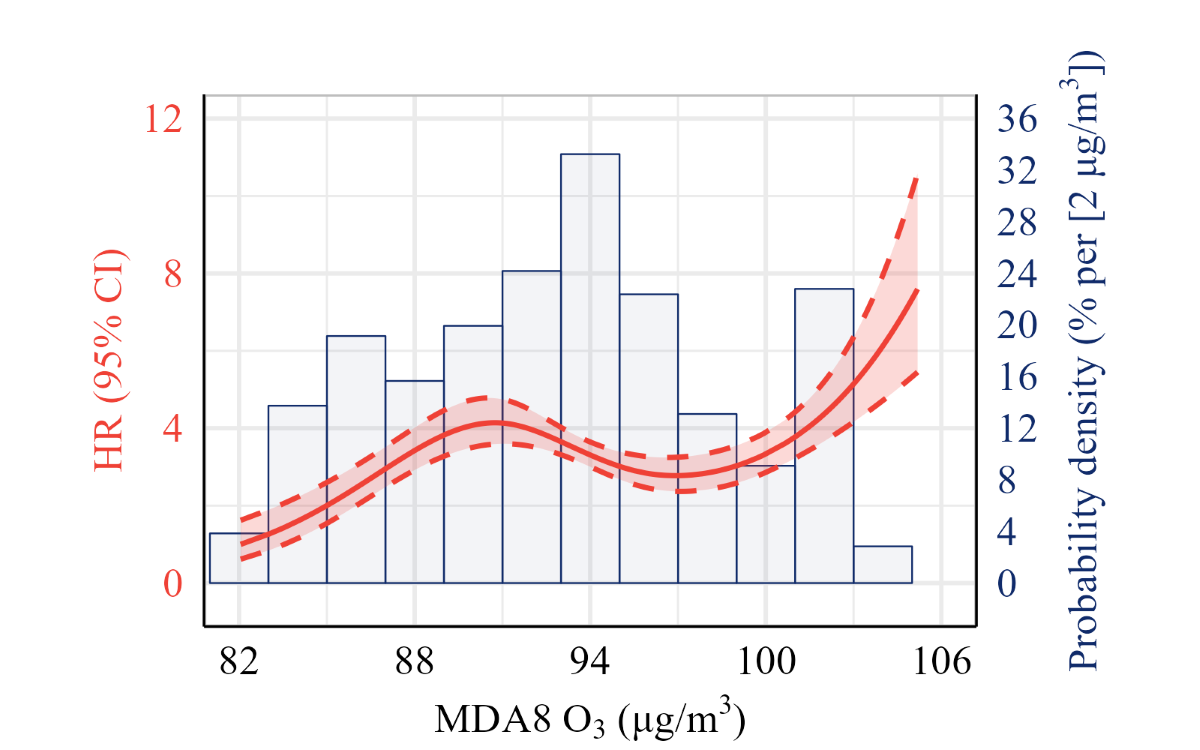
Exposure-response curves of long-term exposure to MDA8 O_3_ concentration with the presence of asthmatic symptoms in China. Adjustment for age, sex, urbanicity, region, smoking status, drinking status, BMI, marriage status, educational level, household income, major type of indoor cooking fuel, and ambient temperature. HR: hazard ratio; MDA8 O_3_: daily 8-hour maximum ozone concentrations.

**Figure 2:**
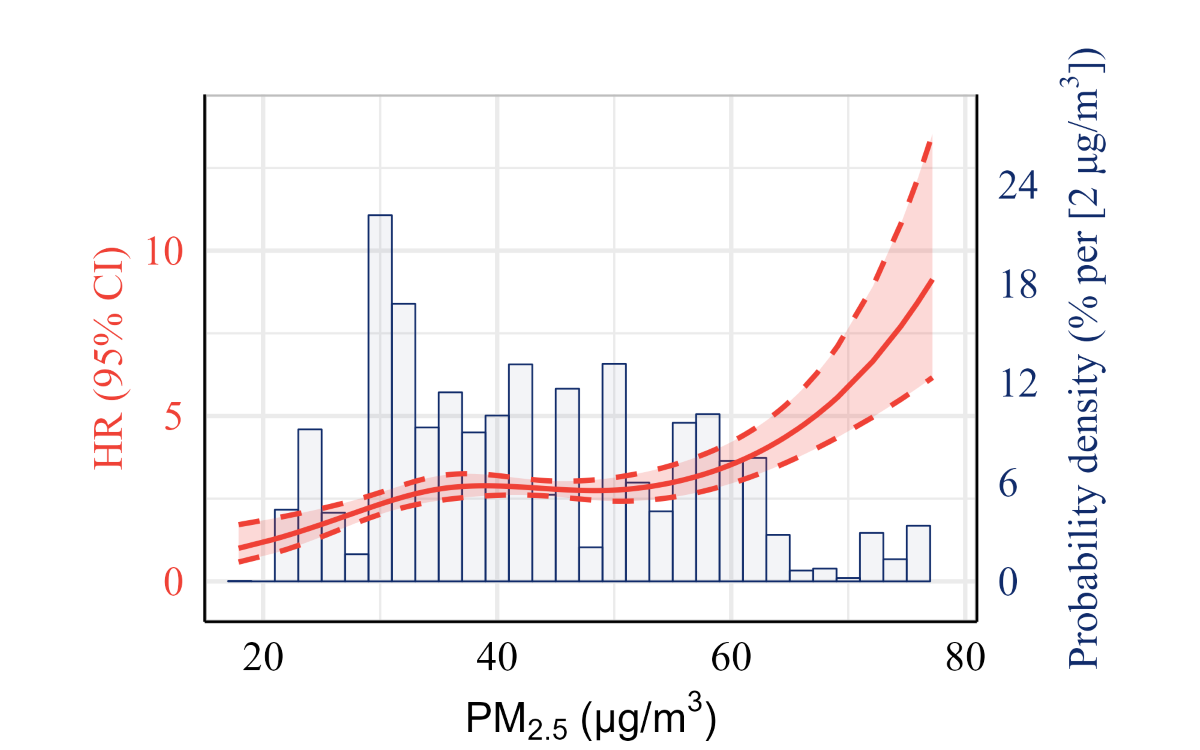
Exposure-response curves of long-term exposure to PM_2.5_ concentration with the presence of asthmatic symptoms in China. Adjustment for age, sex, urbanicity, region, smoking status, drinking status, BMI, marriage status, educational level, household income, major type of indoor cooking fuel, and ambient temperature. HR: hazard ratio; PM_2.5_: particulate matter.

We observed positive nonlinear exposure-response curves of MDA8 O_3_ and PM_2.5_ in the presence of asthmatic symptoms (Figures 1 and 2). Linear analyses showed that per quantile increment of MDA8 O_3_ (HR 1.12, 95% CI 1.01-1.24) and PM_2.5_ (HR 1.18, 95% CI 1.05-1.31) were positively associated with the risk of asthmatic symptoms after adjusted for confounders. Subgroup analyses suggested that the associations were modified by several individual characteristics. For example, the associations between PM_2.5_ and asthmatic symptoms were stronger in rural individuals (HR 1.54, 95% CI 1.30-1.83) than in urban individuals (HR 0.89, 95% CI 0.76-1.05), in individuals with low family income (HR 1.43, 95% CI 1.21-1.69) than in individual with high family income (HR 0.98, 95% CI 0.84-1.14), in individuals using unclean household cooking fuel (HR 1.66, 95% CI 1.38-1.99) than individuals using clean fuel (HR 0.90, 95% CI 0.78-1.04), and in individuals exposed to higher temperatures (HR 1.73, 95% CI 1.27-2.37) than in individuals exposed to lower temperatures (HR 0.92, 95% CI 0.75-1.12; Table 2).

**Table 2:** The individual and joint associations of long-term exposures to MDA8 O_3_ and PM_2.5_ (per quantile increment) in the presence of asthmatic symptoms in China.^a^

Characteristics		Participants (n)	MDA8 O_3,_ HR (95% CI)	*P* for difference test	PM_2.5,_ HR (95% CI)	*P* for difference test	Joint association, HR (95% CI)	*P* for difference test
Total		8490	1.12 (1.01-1.24)	—^b^	1.18 (1.05-1.31)	—	1.18 (1.05-1.33)	—
**Age (years)**								
	50-64	5540	1.09 (0.94-1.26)	—	1.24 (1.06-1.45)	—	1.28 (1.07-1.54)	—
	≥65	2950	1.16 (0.99-1.35)	.58	1.11 (0.95-1.29)	.30	1.04 (0.84-1.27)	.13
**Gender**								
	Male	4066	1.06 (0.92-1.23)	—	1.08 (0.93-1.27)	—	1.19 (1.00-1.42)	—
	Female	4424	1.18 (1.01-1.37)	.35	1.27 (1.08-1.48)	.17	1.17 (0.90-1.51)	.88
**BMI**								
	Normal weight	4683	1.07 (0.94-1.23)	—	1.19 (1.03-1.38)	—	1.14 (0.95-1.37)	—
	Overweight	2772	1.09 (0.90-1.33)	.87	0.98 (0.81-1.19)	.95	1.02 (0.79-1.30)	.37
	Obese	1035	1.87 (1.30-2.69)	.005	1.94 (1.33-2.82)	.20	2.05 (1.32-3.19)	.017
**Urbanicity**								
	Rural	4876	1.15 (1.00-1.32)	—	1.54 (1.30-1.83)	—	1.47 (1.21-1.79)	—
	Urban	3614	1.10 (0.91-1.29)	.69	0.89 (0.76-1.05)	<.001	0.87 (0.70-1.09)	<.001
**Family income**								
	High	4306	1.12 (0.96-1.30)	—	0.98 (0.84-1.14)	—	0.98 (0.80-1.21)	—
	Low	4184	1.14 (0.98-1.32)	.87	1.43 (1.21-1.69)	.001	1.39 (1.13-1.72)	.02
**Major type of household cooking fuel**								
	Clean	4641	1.07 (0.93-1.24)	—	0.90 (0.78-1.04)	—	0.91 (0.77-1.09)	—
	Unclean	3849	1.16 (0.99-1.36)	.47	1.66 (1.38-1.99)	<.001	1.65 (1.29-2.11)	<.001
**Temperature (°C)**								
	Q^c^1 (4.41-12.68)	1931	1.16 (0.95-1.42)	—	1.11 (0.81-1.51)	—	1.16 (0.91-1.48)	—
	Q2 (12.69-15.80)	2140	0.98 (0.81-1.20)	.26	1.18 (0.97-1.44)	.73	1.06 (0.82-1.38)	.49
	Q3 (15.81-16.41)	2071	0.79 (0.67-0.93)	.004	0.92 (0.75-1.12)	.31	0.86 (0.68-1.08)	.10
	Q4 (16.42-22.94)	2348	1.18 (0.87-1.58)	.94	1.73 (1.27-2.37)	.05	1.71 (1.03-2.86)	.30

^a^Adjustment for age, sex, urbanicity, region, smoking status, drinking status, BMI, marriage status, educational level, household income, indoor fuel type, and temperature. In subgroup analyses, other confounders except for the subgroup category variable, analyzed as an independent variable, were adjusted for.

^b^Not applicable.

^c^Q: Quartile.

### Joint Effects of Long-Term Exposure to MDA8 O_3_ and PM_2.5_ With Asthmatic Symptoms

Joint effect analyses showed that per quantile increment of MDA8 O_3_ and PM_2.5_ were associated with an 18% (HR 1.18, 95% CI 1.05-1.33) increase in the risk of asthmatic symptoms. In the joint effect, the contribution of PM_2.5_ and MDA8 O_3_ was 68% and 32%, respectively. We also found greater joint HRs estimated by subgroup analyses in rural residents (HR 1.47, 95% CI 1.21-1.79) than in urban residents (HR 0.87, 95% CI 0.70-1.09), in low-income level (HR 1.39, 95% CI 1.13-1.72) than high-income level (HR 0.98, 95% CI 0.80-1.21), in unclean indoor fuel type (HR 1.65, 95% CI 1.29-2.11) than clean indoor fuel type (HR 0.91, 95% CI 0.77-1.09). Moreover, the highest HR was found in participants with obesity (HR 2.05, 95% CI 1.32-3.19; Table 2).

### PAFs of Asthmatic Symptoms Attributable to MDA8 O_3_ and PM_2.5_

**Figure 3:**
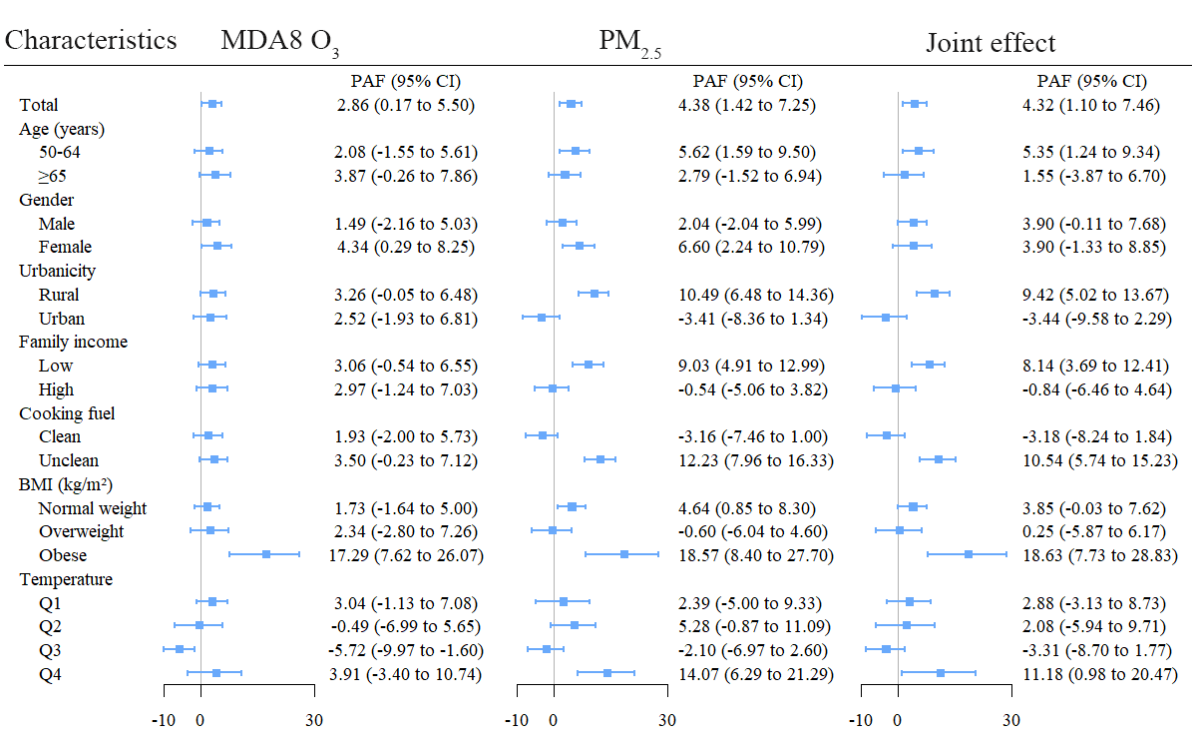
The population-attributable fraction of the presence of asthmatic symptoms attributable to MDA8 O_3_ and PM_2.5_ exposures, estimated by the individual and joint associations. Q: Quartile; MDA8 O_3_: daily 8-hour maximum ozone concentrations; PM_2.5_: particulate matter.

Figure 3 shows the PAFs of asthmatic symptoms attributable to MDA8 O_3_ and PM_2.5_, estimated by the individual and joint associations. In the total participants, the individual PAFs caused by MDA8 O_3_ and PM_2.5_ were 2.86% (95% CI 0.17%-5.50%) and 4.38% (95% CI 1.42%-7.25%), and the joint PAFs by MDA8 O_3_ and PM_2.5_ were 4.32% (95% CI 1.10%-7.46%). Subgroup analyses showed a large variation of PAFs among different groups. For example, the PAFs of asthmatic symptoms were the largest in obese individuals. The individual PAF caused by MDA8 O_3_ and PM_2.5_ was 17.29% (95% CI 7.62%-26.07%) and 18.57% (95% CI 8.40%-27.70%), and the joint PAFs by MDA8 O_3_ and PM_2.5_ was 18.63% (95% CI 7.73%-28.83%). More detailed information is shown in Figure 3.

### Sensitivity Analyses

Results indicated that HRs did not substantially change after adjustment for different confounders (Table S4 in Multimedia Appendix 1).

## Discussion

### Principal Findings

In this national cohort study, we found a positive correlation between individual and joint associations on long-term exposure to ambient PM_2.5_ and MDA8 O_3_ with the risk of asthmatic symptoms in a Chinese population older than 50 years. Based on the joint associations, 4.32% of asthmatic symptoms could be attributable to PM_2.5_ and MDA8 O_3_ exposures, and ambient PM_2.5_ contributed much more to the joint effect. In addition, the associations were modified by obesity, urbanicity, family income level, household cooking fuel, and ambient temperature. Our findings are significant for estimating the disease burden of air pollution and making policies for air pollution control.

In the literature, relatively fewer studies have investigated the associations of long-term exposure to PM_2.5_ and MDA8 O_3_ with asthma incidence in adults compared to those studies on short-term exposures and on childhood asthma. Some studies reported consistent results with this study that long-term exposure to PM_2.5_ and MDA8 O_3_ were separately associated with an increased risk of asthma incidence [6-8]. For example, the Health Effects Institute reported that long-term exposure to higher PM_2.5_ was associated with an increased risk of asthma, which was from the European Cohorts in the ELAPSE Project [6]. The Epidemiological Study of the Genetic and Environmental Factors of Asthma reported that the risk of asthma increased with O_3_ exposure [7]. McDonnell et al [39] also reported that long-term exposure to O_3_ was positively associated with new-onset asthma in adult males. However, several previous studies did not find positive associations of PM_2.5_ and MDA8 O_3_ with the incidence of asthma [12,13,40]. For example, a prospective cohort from Australia reported a null association between long-term exposure to PM_2.5_ with adult-onset asthma [12]. The results from the ELAPSE project reported a negative association (HR 0.90, 95% CI 0.81-0.99 for each 10 μg/m^3^) between long-term exposure to O_3_ and the incidence of asthma [6]. Although it may be difficult to directly compare the results among these studies due to different study populations, this interstudy variation of associations suggests that the associations of long-term exposure to PM_2.5_ and O_3_ with asthma incidence remain inconclusive, and more studies are needed particularly in low-and middle-income countries. For example, we did not find a prospective cohort that has investigated the associations of long-term exposure to PM_2.5_ and O_3_ with asthma in China.

Exposure to air pollution can trigger inflammatory and immune responses, oxidative stress, and airway remodeling in the development and exacerbation of asthma. Exposure to PM_2.5_ induces the release of inflammatory mediators from alveolar macrophages, which is an important pathogenesis of pulmonary inflammation in the development of asthma. PM_2.5_ induces inflammatory responses, associated with Th1/Th2 pathway imbalance and leads to Th2-oriented inflammation [41]. Apart from that, PM_2.5_ induces the production of reactive oxygen species. Reactive oxygen species will further enhance the oxidative stress response, resulting in DNA, protein, lipid, and other cellular and molecular damage, leading to respiratory disease [42,43]. In this study, PM_2.5_ also induced a significant upregulation of vascular endothelial growth factor A production, a signaling event that controls vascular remodeling [44]. Furthermore, exposure to O_3_ is associated with an increase in free radicals and biomarkers associated with oxidative stress [45]. Ozone exposure also induced airway inflammation with increased numbers of neutrophils [46].

More importantly, we quantified the joint associations of long-term exposure to PM_2.5_ and MDA8 O_3_ in the presence of asthmatic symptoms in a Chinese population. Our findings had 2 major implications. First, PM_2.5_ (68%) contributed more to the joint associations than O_3_ (32%), suggesting the more important role of PM_2.5_ in the development of asthmatic symptoms. Second, the PAFs of asthmatic symptoms based on the joint associations were lower than the sum of PAFs based on the individual associations, which suggests that the calculation based on a single-pollutant model might overestimate the effects of air pollutants due to the potential mutual confounding [47]. Several studies reported similar findings to this study [47-49]. For example, Winquist et al [47] reported smaller joint effect estimates from multipollutant models than estimates from single-pollutant models. In practice, people are usually exposed to multiple pollutants as air pollution complex mixture. Therefore, our findings combined with previous studies suggested that estimating the disease burden of air pollution calculated using a joint effect model may be more appealing because it transparently and explicitly specifies the contribution of various pollutants of mixtures.

PAF is a statistical indicator that quantitatively describes the health impact of a certain risk factor on a population. Specifically, it represents the proportion of total disease (or mortality) in the population that can be attributed to a certain factor, so we describe that a total of 4.32% of the presence of asthmatic symptoms could be attributable to joint PM_2.5_ and MDA8 O_3_ exposures. Although this figure is not very large, air pollution exposure is ubiquitous, and the large number of patients with asthmatic symptoms in China may indicate important public health issues. It was estimated that there were about 848,000 incident cases with asthmatic symptoms in 2019 in Chinese people older than 50 years [2]. As a result, there were about 37,000 cases with asthmatic symptoms, which may have been caused by exposure to PM_2.5_ and O_3_.

The results of stratified analyses showed greater effects of PM_2.5_ and O_3_ on the risk of asthmatic symptoms in participants in rural areas than in urban areas. The stronger associations in rural areas may be because the rural population is more likely to smoke and use unclean cooking fuel. Usage of unclean cooking fuel could also increase the risk of asthmatic symptoms through similar mechanisms of air pollution [50,51], and hence synergize the effects of ambient air pollutants. Compared with urban residents, rural residents usually have harder physical activities, which may increase their exposure to air pollution. Moreover, the rural population may be more susceptible to air pollution due to the disadvantaged sanitation and health care systems, dwelling environments, poor health status, and so forth [52]. These potential mechanisms were also confirmed by our findings of greater associations in participants with lower family income and using unclean energy as the major household cooking fuel.

The stratified analyses also showed greater associations in obese participants than in lean individuals. The modification of obesity on the respiratory health impacts of air pollution has been reported in other studies [53,54]. For example, a cross-sectional study in northeastern Chinese cities reported that obesity enhanced the respiratory health effects caused by air pollution in children [53]. Obesity is a proinflammatory state, and the adipose tissue propagates inflammation by recruitment of macrophages via chemokines such as monocyte chemoattractant protein-1, and via elaboration of cytokines and chemokines such as leptin, interleukin-6, and tumor necrosis factor α. Meanwhile, inhaled air pollutants could also produce these cytokines and chemokines, and hence increase inflammation [53,54]. As a result, obesity may amplify the inflammatory response induced by air pollution and increase the vulnerability of obese participants to harmful respiratory effects caused by air pollution exposures.

There are a few limitations in this study. First, many participants were lost to follow-up due to reasons such as immigration, urbanization, and so forth, which may lead to selection bias. Second, the presence of asthmatic symptoms was based on self-reported questionnaires, which may lead to misclassification bias. However, all reported patients must be diagnosed by a doctor or by clinical symptoms, which could partially reduce the bias. Third, some potential confounders such as physical activity, occupational exposure to air pollution, vegetable and fruit intake, and the higher rate of missing information were not adjusted for in the main models. However, the results of sensitivity analyses conducted only in those participants with complete information showed that the results are robust to these factors (Table S4 in Multimedia Appendix 1). Fourth, we did not include other air pollutants such as NO_2_, SO_2_, and CO due to the unavailability of data in the earlier years. In particular, NO_2_ was involved in the production of atmospheric ozone, and the effects of MDA8 O_3_ were biased due to the unavailability of NO_2_. Therefore, more studies are needed in the future. Fifth, 2 different methods were applied to assess the participants’ exposure to ambient MDA8 O_3_ and PM_2.5_, which may affect the result. However, the 2 methods are commonly adopted at present in air pollution exposure assessment, and the prediction results of the 2 models have been verified with high accuracy, which suggests that the impacts of using different assessment methods on our findings may be limited.

### Conclusions

In conclusion, this study provided novel evidence that long-term exposures to ambient PM_2.5_ and O_3_ were individually and jointly associated with a higher presence of asthmatic symptoms, and ambient PM_2.5_ contributed more to the joint effects. The combined effects of air pollutants based on a single-pollutant model might be overestimated. The joint effects were more pronounced in participants with obesity, from rural areas, with lower income levels, and who use unclean household cooking fuel.

## Appendix

Multimedia Appendix 1 The joint effects of long-term exposure to ambient PM_2.5_ and ozone on incident asthma: prospective cohort study.

## Abbreviations

COPD: chronic obstructive pulmonary disease
GBD: Global Burden of Disease
HR: hazard ratio
MDA8 O_3_: daily 8-hour maximum ozone concentrations
PAF: population-attributable fraction
PM_2.5_: particulate matter
WHO SAGE: World Health Organization Study on Global Ageing and Adult Health

## Data Availability

The data that support the findings of this study are available on request from the corresponding author.
