# Supplementary material for: Joint Effects of Long-Term Exposure to Ambient Fine Particulate Matter and Ozone on Asthmatic Symptoms: Prospective Cohort Study
Source: JMIR Public Health Surveill. 2023 Aug 3;9:e47403. doi: 10.2196/47403 (PMC10436124; doi:10.2196/47403)
Supplement: Multimedia Appendix 1 [file publichealth_v9i1e47403_app1.docx]

**Figure and table of contents**

**Figure S1.** The selection process of study participants.

**Table S1**. The definition of covariates.

**Table S2.** VIFs among single pollutant model and joint effects model.

**Table S3.** The correlations among annual average PM_2.5_, MDA8 O_3_ and temperature.

**Table S4**. Sensitivity analyses of using different models and confounders adjustment on the associations of MDA8 O_3_ and PM_2.5_ with asthma.

**Figure S2.** Exposure-response curves of long-term exposure to PM_2.5_ concentration (per quantile) with the presence of asthmatic symptoms in China.

**Figure S3.** Exposure-response curves of long-term exposure to MDA8 O_3_ concentration (per quantile) with the presence of asthmatic symptoms in China.

**Figure S1.** The selection process of study participants.

33 participants with unavailable year of follow-up were excluded

8,053 participants with no follow-up information were excluded

676 participants without information of asthma were excluded

18,673 participants were initially recruited

420 participants with asthma at baseline were excluded

728 participants with age<50 years at baseline were excluded

8,490 participants were finally included in the analysis

273 participants without information of covariates were excluded

**Table S1**. The definition of covariates.

|  | Category | Definition |
| --- | --- | --- |
| Asthma | Yes | If they answered ‘yes’ to these questions: “During the last 12 months, have you experienced attacks of wheezing or whistling breathing?”(1), “During the last 12 months, have you experienced attack of wheezing that came on after you stopped exercising or some other physical activity?”(2), “During the last 12 months, have you experienced a feeling of tightness in your chest?”(3), “During the last 12 months, have you woken up with a feeling of tightness in your chest in the morning or any other time?”(4), “During the last 12 months, have you had an attack of shortness of breath that came on without obvious cause when you were not exercising or doing some physical activity?” (5). A respondent was considered to have asthma if he/she answered yes to question (1) and yes to any of the follow-up questions (2-5). |
|  | No | Participants who does not meet the criteria of ‘yes’. |
| Vegetable and fruit intake | Sufficient | Intaking more than or equal to five servings based on the consumption per day |
|  | Insufficient | Intaking less than five servings based on the consumption per day |
| Household income | High | More than or equal to median |
|  | Low | Less than median |
| Major type of household cooking fuel | Clean | Including electricity and natural gas |
|  | Unclean | Including coal, wood, dung, and agricultural residues |
| Physical activity[1] | High | (1) At least three days of vigorous-intensity activity with a minimum of 1500 Metabolic equivalent (MET) minute per week or seven or more days of any combination of walking, moderate-intensity or (2) vigorous-intensity activities with a minimum of 3000 MET-minute per week |
|  | Middle | (1) Three or more days of vigorous-intensity activity of at least 20 minutes per day or five or more days of moderate-intensity activity or (2) at least 30 minutes of walking per day or five or more days of any combination of walking, moderate or vigorous-intensity activities with a minimum of 600 MET-minute per week |
|  | Low | Participants who does not meet any of the criteria of high and low |
| Marriage status | Married | Including currently married and cohabiting |
|  | Unmarried | Including never married, separated, divorced, and widowed |

**Table S2.** VIFs among single pollutant model and joint effects model.

|  | PM_2.5_ | MDA8 O_3_ | Temperature |
| --- | --- | --- | --- |
| PM_2.5_ and MDA8 O_3_ with joint effects model | 2.259 | 2.667 | 5.644 |
| PM_2.5_ with single pollutant model | 1.601 |  | 2.754 |
| MDA8 O_3_ with single pollutant model |  | 1.92 | 5.093 |

**Table S3.** The correlations among annual average PM_2.5_, MDA8 O_3_ and temperature.

|  | PM_2.5_ | MDA8 O_3_ | Temperature |
| --- | --- | --- | --- |
| PM_2.5_ (μg/m^3^) | 1 | 0.663 | 0.090 |
| MDA8 O_3_ (μg/m^3^) |  | 1 | 0.428 |
| Temperature (℃) |  |  | 1 |

The p-values of all correlation coefficients <0.001.

**Table S4**. Sensitivity analyses of using different models and confounders adjustment on the associations of MDA8 O_3_ and PM_2.5_ with asthma.

| Model | n | HR (95%CI) for per quantile increment in MDA8 O_3_ | HR (95%CI) for per quantile increment in PM_2.5_ |
| --- | --- | --- | --- |
| Model 1 | 8490 | 1.14(1.05, 1.24) | 1.27(1.15, 1.39) |
| Model 2 | 8490 | 1.05(0.97,1.14) | 1.17(1.05, 1.31) |
| Model 3 | 8490 | 1.12(1.01,1.24) | 1.18(1.07,1.29) |
| Model 4 | 8490 | 1.10(0.99,1.22) | 1.42(1.29,1.58) |
| Model 5 | 7373 | 1.13(1.00,1.27) | 1.22(1.08,1.37) |
| Model 6 | 7373 | 1.14(1.01,1.28) | 1.22(1.09,1.38) |
| Model 7 | 6144 | 1.14(1.00,1.29) | 1.27(1.12,1.45) |
| Model 8 | 6144 | 1.14(1.00,1.30) | 1.28(1.12,1.46) |
| Model 9 | 8490 | 1.05(0.89,1.22) | 1.56(1.39,1.85) |

Model 1: Unadjusted;

Model 2: Adjustment for age, sex, urbanicity, region, smoking status, drinking status, BMI, marriage status, educational level, household income, indoor fuel type;

Model 3: Adjustment for covariates in Model 2 and temperature (fully model);

Model 4: Fully model + adjustment for the time of population entering the cohort;

Model 5: Fully model + adjustment for physical activity, which was analyzed in participants with complete data.

Model 6: Fully model + without adjustment for physical activity, which was analyzed in participants with complete data.

Model 7: Fully model + adjustment for physical activity, fruit intake, vegetable intake, and air pollution related occupations, which was analyzed in participants with complete data.

Model 8: Fully model + without adjustment for physical activity, fruit intake, vegetable intake, and air pollution related occupations, which was analyzed in participants with complete data.

Model 9: Fully model with air pollution of average exposure from baseline to final wave.

**Figure S2.** Exposure-response curves of long-term exposure to PM_2.5_ concentration (per quantile) with the presence of asthmatic symptoms in China.

**
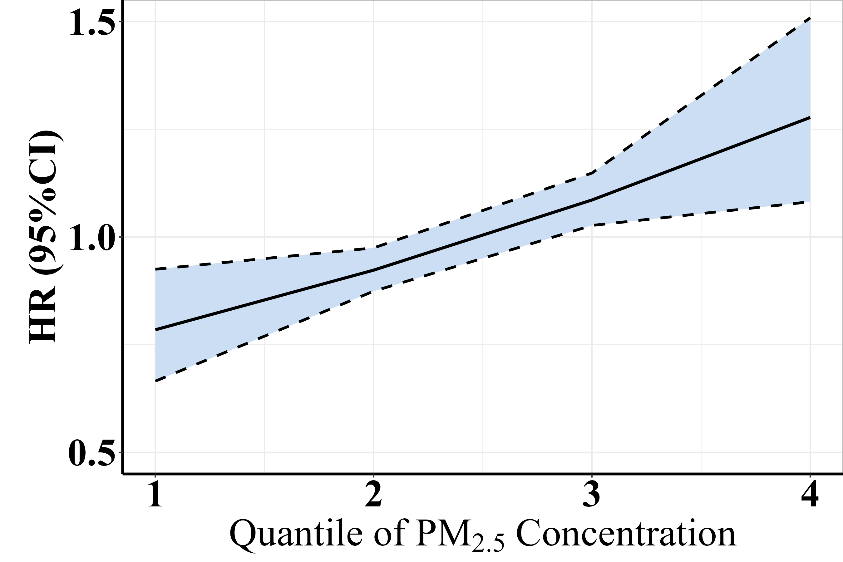
**

**Figure S3.** Exposure-response curves of long-term exposure to MDA8 O_3_ concentration (per quantile) with the presence of asthmatic symptoms in China.

**
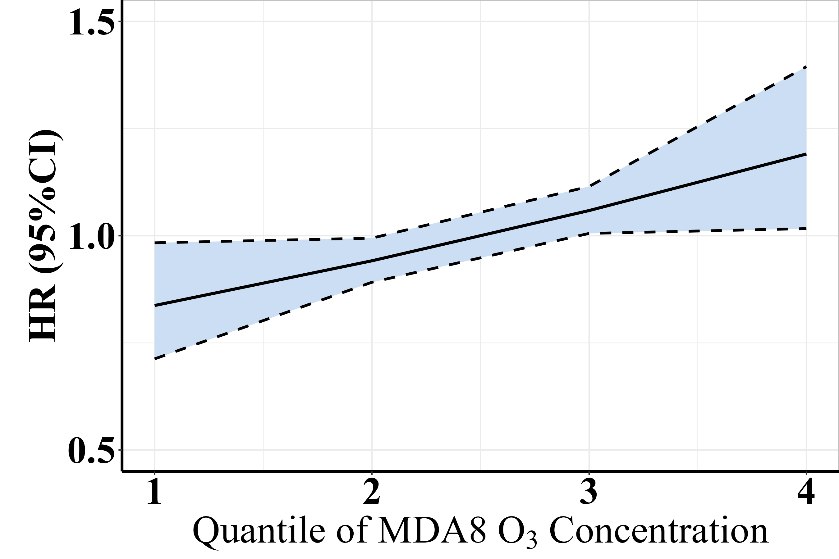
**

**References**

1. Chu AH, Ng SH, Koh D, Müller-Riemenschneider F. Reliability and Validity of the Self- and Interviewer-Administered Versions of the Global Physical Activity Questionnaire (GPAQ). PLoS One 2015;10(9):e0136944. PMID: 26327457. doi: 10.1371/journal.pone.0136944.
